# Traditional Herbal Medicine Use Associated with Liver Fibrosis in Rural Rakai, Uganda

**DOI:** 10.1371/journal.pone.0041737

**Published:** 2012-11-27

**Authors:** Brandon J. Auerbach, Steven J. Reynolds, Mohammed Lamorde, Concepta Merry, Collins Kukunda-Byobona, Ponsiano Ocama, Aggrey S. Semeere, Anthony Ndyanabo, Iga Boaz, Valerian Kiggundu, Fred Nalugoda, Ron H. Gray, Maria J. Wawer, David L. Thomas, Gregory D. Kirk, Thomas C. Quinn, Lara Stabinski, Rakai Health Sciences Program

**Affiliations:** 1 Infectious Diseases Institute, College of Health Sciences, Makerere University, Kampala, Uganda; 2 Harvard Medical School, Boston, Massachusetts, United States of America; 3 Division of Intramural Research, National Institute of Allergy and Infectious Diseases, National Institutes of Health, Bethesda, Maryland, United States of America; 4 Division of Infectious Diseases, Department of Medicine, School of Medicine, Johns Hopkins University, Baltimore, Maryland, United States of America; 5 Department of Pharmacology and Therapeutics, School of Medicine, University of Dublin, Trinity College, Dublin, Ireland; 6 St James's Hospital, Dublin, Ireland; 7 Department of Medicine, College of Health Sciences, Makerere University, Kampala, Uganda; 8 Department of Botany, Makerere University, Kampala, Uganda; 9 Rakai Health Sciences Program, Entebbe, Uganda; 10 Department of Population, Family, and Reproductive Health, Bloomberg School of Public Health, Johns Hopkins University, Baltimore, Maryland, United States of America; 11 Department of Epidemiology, Bloomberg School of Public Health, Johns Hopkins University, Baltimore, Maryland, United States of America; Saint Louis University, United States of America

## Abstract

**Background:**

Traditional herbal medicines are commonly used in sub-Saharan Africa and some herbs are known to be hepatotoxic. However little is known about the effect of herbal medicines on liver disease in sub-Saharan Africa.

**Methods:**

500 HIV-infected participants in a rural HIV care program in Rakai, Uganda, were frequency matched to 500 HIV-uninfected participants. Participants were asked about traditional herbal medicine use and assessed for other potential risk factors for liver disease. All participants underwent transient elastography (FibroScan®) to quantify liver fibrosis. The association between herb use and significant liver fibrosis was measured with adjusted prevalence risk ratios (adjPRR) and 95% confidence intervals (CI) using modified Poisson multivariable logistic regression.

**Results:**

19 unique herbs from 13 plant families were used by 42/1000 of all participants, including 9/500 HIV-infected participants. The three most-used plant families were Asteraceae, Fabaceae, and Lamiaceae. Among all participants, use of any herb (adjPRR = 2.2, 95% CI 1.3–3.5, p = 0.002), herbs from the Asteraceae family (adjPRR = 5.0, 95% CI 2.9–8.7, p<0.001), and herbs from the Lamiaceae family (adjPRR = 3.4, 95% CI 1.2–9.2, p = 0.017) were associated with significant liver fibrosis. Among HIV infected participants, use of any herb (adjPRR = 2.3, 95% CI 1.0–5.0, p = 0.044) and use of herbs from the Asteraceae family (adjPRR = 5.0, 95% CI 1.7–14.7, p = 0.004) were associated with increased liver fibrosis.

**Conclusions:**

Traditional herbal medicine use was independently associated with a substantial increase in significant liver fibrosis in both HIV-infected and HIV-uninfected study participants. Pharmacokinetic and prospective clinical studies are needed to inform herb safety recommendations in sub-Saharan Africa. Counseling about herb use should be part of routine health counseling and counseling of HIV-infected persons in Uganda.

## Introduction

Traditional herbal medicines are commonly used for HIV/AIDS and other health conditions in Uganda and sub-Saharan Africa, often in parallel with programs that provide antiretroviral therapy (ART). In the 1990's an estimated 80% of Ugandans living in rural villages used traditional healers for primary health care [1]. A study of 137 HIV-infected Ugandans receiving ART found that 60% used herbs concurrently with ART [2].

In Uganda traditional herbal medicines are usually boiled extracts of herbs taken orally [3]. Some potentially hepatotoxic traditional herbal medicines used in Uganda and sub-Saharan Africa include *Hoodia gordoni* [4], kava [5], *Phytolacca dioica* [6], and herbs from the Asteraceae family [7]. Little is known about the hepatotoxicity of other commonly used herbs or the contribution of herbs to the burden of liver fibrosis and hepatocellular carcinoma in sub- Saharan Africa, including when used concomitantly with ART. Data on the specific types of herbs taken by HIV-infected persons in Uganda is limited, as is information about their components, side effects, toxicities, and ART interactions [8].

In Rakai, Uganda, liver toxicity associated with herbal medicine may be of particular concern given the high prevalence of significant liver disease (17%) among HIV-infected persons in Rakai recently identified by transient elastography (FibroScan®, Echosense, Paris, France) [9]. In the aforementioned study, reported herbal medicine use was associated with a two-fold increased risk of significant liver disease, defined as a transient elastography score equivalent to METAVIR liver fibrosis stage 2 (portal fibrosis with few septa) or greater [9]. The study presented here follows up on this prior investigation with an in-depth analysis of the herbs used by study participants and their relation to liver fibrosis.

## Methods

This cross-sectional study enrolled 500 HIV-infected participants receiving care at five HIV care clinics within the Rakai Health Sciences Program (RHSP) HIV Care Program. 500 HIV- uninfected participants from the Rakai Community Cohort Study (RCCS) were frequency- matched to these participants by age, gender, and community. Begun in 1994 in one of Uganda's hardest-hit regions by the HIV epidemic, the RCCS conducts annual surveys in a population of 10,000–15,000 people aged 15–49 years, and is described in detail elsewhere [10]. Participants underwent a detailed liver-disease focused risk factor questionnaire which included an assessment of herbal drug use, venous blood collection, and transient elastography (FibroScan®, Echosense, Paris, France) to quantify liver fibrosis.

### Ethics Statement

Written informed consent was obtained from all participants. Institutional Review Boards of theNational Institute of Allergy and Infectious Diseases, the Johns Hopkins Medical Institutions, the Scientific and Ethics Committee of the Uganda Virus Research Institute, and the Uganda National Council for Science and Technology approved this study. The study protocol conforms to the ethical guidelines of the 1975 Declaration of Helsinki, and is registered on clinicaltrials.gov (#NCT00782158).

### Herb Use Assessment

Participants were asked about any current herb use and then to name the two herbs they used most often. Scientific names were assigned to local herb names in consultation with local traditional medicine practitioners and a member of Makerere University Botany Department (CKB). The Makerere University Herbarium database was also used to validate herb identities. Some participants reported non-plant substances such as clay and spiritual charms as herb use. Participants reporting use of non-plant based entities were reclassified as non-herb users in this analysis.

### Laboratory Assays

HIV-1 serology was determined by two HIV-1 enzyme immuno-assays: Vironostika HIV-1 (OrganonTeknika, Charlotte, North Carolina, USA) and Cambridge Biotech (Worcester, Massachusetts, USA). Participants with discrepant HIV-1 enzyme immune assay results were tested with western blot (HIV-1 Western Blot; Bio-Merieux-Vitek, St. Louis, Missouri, USA). For HIV-infected participants, current CD4 count (within 12 months) and CD4 count nadir were abstracted from the RHSP HIV Care Program database. CD4 counts were measured by FACSCalibur flow cytometer (software version 1.4, Becton Dickinson, San Jose, California, USA). Hepatitis B virus surface antigen (HBsAg) was determined using ELISA (ETI-MAK-2 Plus, Diasorin, Vercelli, Italy). Alanine aminotransferase (ALT) was tested using standard methods (COBAS CII; Roche, Basel, Switzerland), and hepatotoxicity was defined by ALT elevations and classified according to AIDS Clinical Trial Group criteria [11]. The upper limit of normal for ALT was defined as 19 IU/L in women and 39 IU/L in men [12], [13].

### Transient Elastography

Transient elastography or FibroScan® is a novel, validated, noninvasive technology for the evaluation of fibrosis in chronic liver disease [14]. A FibroScan® is approximately the size of an ultrasound unit. A probe placed over a patient's abdomen produces vibration and the speed of the responding elastic wave is detected by ultrasound. The propagation of these waves through the liver is directly correlated to the degree of liver stiffness. The results are instantaneously received as a single, quantitative parameter of liver stiffness measurement (LSM, reported in kPa). Each transient elastography scan takes ten liver stiffness measurements in rapid succession over several seconds. The median of the ten measurements is reported as the final liver stiffness measurement. The procedure is non-invasive, painless, has no side effects, and requires only a few minutes to perform. The device requires minimal training and does not need to be performed by advanced medical personnel.

In this study a conservative liver stiffness measurement cutoff of ≥9.3 kPa, from a validation study in persons of predominantly African descent, was used to define significant fibrosis equivalent to METAVIR fibrosis stage 2 (portal fibrosis with few septa) or greater [15]. Two study nurses at the Rakai Health Science Program study site conducted all transient elastography scans after receiving certification from the manufacturer. According to manufacturer recommendations, scans with high variability—defined as an interquartile range greater than 30% of the median LSM value from an individual examination—were not considered valid and were excluded from the analysis. Participants with invalid scans on an initial attempt were repositioned and rescanned up to 4 times to achieve a valid scan.

### Statistics

Baseline demographic, behavioral and clinical characteristics were compared by HIV status. Differences in continuous variables were assessed using t-tests and Wilcoxon-Mann-Whitney tests. Categorical variables were compared using Pearson's chi squared test.

The primary outcome measure was liver fibrosis. Because odds ratios may overestimate the magnitude of association between variables if the outcome of interest is common, adjusted prevalence risk ratios (adjPRR) with 95% confidence intervals (95% CI) were estimated using modified Poisson regression [16]. The multivariable models adjusted for HIV, gender, occupation in the fishing industry, chronic hepatitis B infection (positive hepatitis B surface antigen), and drinking ≥1.25 liters per week of liquor, as these risk factors were associated with liver disease in previous analysis of this study population [9]. Age was included in all models and nadir CD4 cell count and ART status were included in models restricted to HIV- infected participants for reasons of biologic plausibility. STATA version 11.0 (STATA Corp, College Station, TX) was used for statistical analysis.

## Results

### Baseline Characteristics

The HIV-infected and uninfected groups each had 67% females (see table 1). The median age of 38 years in the HIV-infected group was close to the median age of 37 years in the HIV- uninfected group (p = 0.025). Only 2% of both HIV-infected and uninfected participants were heavy liquor drinkers (p = 0.65). The prevalence of chronic HBV infection was similar in both groups, 5% in HIV-infected participants and 3% in HIV-uninfected participants (p = 0.010). 29% of HIV-infected participants and 17% of HIV-uninfected participants had any grade 1 or higher hepatotoxicity by AIDS Clinical Trial Group (ACTG) criteria (p<0.001). No participants demonstrated grade 4 hepatotoxicity.

**Table 1:** Baseline characteristics of study participants.

	HIV-infected pts (n = 500)	HIV-uninfected pts (n = 500)	
	n (% or IQR)	n (% or IQR)	p value
**Characteristic**			
Median Age, years	38 (IQR 31–44)	37 (IQR 32–44)	0.025
Female	312 (67%)	333 (67%)	0.89
Heavy Liquor use (>1.25 L/week)	11 (2%)	9 (2%)	0.65
Lifetime occupational fishing	5 (1%)	1 (0.2%)	0.65
HBsAg positive	23 (5%)	14 (3%)	0.10
Valid TE scan	468 (94%)	494 (99%)	<0.001
**Herb Use**			
Current herb use	8 (2%)	33 (7%)	0.0001
Known herbs	5 (0.9%)	16 (3%)	0.015
Unknown herbs	4 (0.9%)	17 (3%)	0.004
Asteraceae family	2 (0.4%)	6 (1%)	0.16
Fabaceae family	0 (0%)	6 (1%)	0.014
Lamiaceae family	1 (0.2%)	4 (0.8%)	0.18
**ACTG Heptatotoxicty Criteria**			
Median ALT (U/L)	22 (IQR 16–31)	19 (IQR 15–25)	<0.001
Grade 0 (<1.25×ULN) by ALT	354 (71%)	414 (83%)	
Grade 1 (1.25–2.5×ULN) by ALT	122 (24%)	77 (15%)	
Grade 2 (2.6–5×ULN) by ALT	19 (4%)	9 (2%)	
Grade 3 (5.1–10×ULN) by ALT	5 (1%)	0 (0%)	
Grade 4 (>10×ULN) by ALT	0 (0%)	0 (0%)	
**CD4 and ART Characteristics**			
Current CD4 count (cells/uL)	449 (IQR 320–642)		
Nadir CD4 count (cells/uL)	214 (IQR 130–350)		
Nadir CD4 count <100 cells/uL	95 (19%)		
On ART	302 (60%)		
ART duration (months)	19 (IQR 9–38)		

HIV (Human Immunodeficiency Virus), IQR (Interquartile Range), HBsAg (Hepatitis B Surface Antigen), ACTG (AIDS Clinical Trials Group), CD4 (Cluster of Differentiation 4 positive Helper T cells), ART (Antiretroviral Therapy).

At the time of enrolment HIV-infected participants had a median CD4 count of 449 cells/µL (IQR 320–642) and 60% were receiving ART with a median duration of 19 months (IQR 9–38). Demographics of the HIV-infected group were also similar to participants in the Rakai Health Sciences HIV Care Program, in which 65% of participants are female, 64% are on ART, and the median CD4 count is 480 cells/µL.

468/500 (94%) of HIV-infected participants and 494/500 (99%) of HIV-uninfected participants had valid elastography scans. Those with valid scans were included in the assessment of liver fibrosis and were included in the regression models.

### Characteristics of Herb Users

42/1000 (4%) of all participants reported current use of traditional herbal medicines, including 9/500 (2%) of HIV-infected participants and 33/500 (7%) HIV-uninfected participants (table 1). 21/42 (50%) of participants could name at least one herb they were taking. 4/46 (9%) of participants reporting herb use were reclassified as not taking herbs because they only reported use of inert, non-plant substances including clay. Herb users did not differ by age (p = 0.61) or gender (p = 0.15) from those who did not report herb use (see table 2). Herb users were not more likely to work in the fishing industry (p = 0.13) or have chronic hepatitis B infection (p = 0.73). 7% of participants reporting herb use drank liquor heavily (≥1.25 L/week), compared to 2% of participants who did not report herb use (p = 0.015). 19 unique herbs from 13 families were used, and are characterized in tables 3 and 4. The most common families were Asteraceae, Fabaceae, and Lamiaceae, which were used by eight, six and five participants, respectively.

**Table 2:** Characteristics of participants reporting current herb use.

	Using Herbs (n = 42)	Not Using Herbs (n = 958)	
Characteristic	n (% or IQR)	n (% or IQR)	p value
Median Age, years	39 (32–44 IQR)	38 (31–44 IQR)	0.61
Female	27 (64%)	643 (67%)	0.15
Heavy Liquor use (>1.25 L/week)	3 (7%)	17 (2%)	0.015
Lifetime occupational fishing	1 (2%)	5 (0.5%)	0.13
HBsAg positive	2 (5%)	35 (4%)	0.73
HIV infected	9 (20%)	491 (50%)	0.0001

L (Liters), HBsAg (Hepatitis B Surface Antigen), HIV (Human Immunodeficiency Virus).

**Table 3:** Characteristics of known herbs in the Asteraceae, Fabaceae, and Lamiaceae families.

Family (n taking)	Scientific Name	Local Names (n taking)	English Name	Taken in Rakai for:	Known Pharmacology	Known Liver Toxicity and/or ART Interaction
Asteraceae (8)	*Vernonia amygdalina*	Mululuza (4)	Bitter leaf	Fever, fever with jaundice	Contains alkaloids, saponins, tannins, flavonoids, steroid glycosides, sesquiterpine lactone [32]	Hepatotoxic at high doses (750 mg/kg) [24]. One herb from the Veronia genus (V. lasiopus) was hepatotoxic in an in-vitro rat precision cut liver slice model [33]. Many herbs in the Asteraceae family contain pyrrolizidine alkaloids, which are associated with veno-occlusive liver disease [17]
	*Vernonia* genus	Kiluluuza (2)				
	*Microglossa densiflora*	Kafugankande, Akafugankande (1)		Fever, indigestion, loose stools, parasites	Microglossa family contains clerodane diterpenoids [34]	A similar herb from the Microglossa family (M. *pyrifolia*) was hepatotoxic in an *in-vitro* rat precision cut liver slice model [33]. Many herbs in the Asteraceae family contain pyrrolizidine alkaloids, which are associated with veno-occlusive liver disease [17]
	*Aspilia africana*	Makaayi (1)	Wild sunflower	Fever with jaundice	Contains saponins, tannins, terpenoids, Sesquiterpenes, monoterpenes [35], [36]	No hepatoxicity in rat in vivo model [38]. Many herbs in the Asteraceae family contain pyrrolizidine alkaloids, which are associated with veno-occlusive liver disease [17]
Fabaceae (6)	*Pseudarthria hookeri*	Bikakala, Kikakala, Omukakala, Mukakala (4)		Fever, fever with jaundice, allergy, cough, wounds	May have estrogenic activity [37]	Many herbs in the Fabaceae family contain pyrrolizidine alkaloids, which are associated with veno-occlusive liver disease [17]
	*Indigofera congesta*	Namasumi (2)	Indigo	Fever with jaundice, antenatal health	Indigofera family members contain flavonoids, saponins, quinones, sterols/triterpenes, tannins, gallic acid, caffeic acid, rutin and myricetin [38]	Many herbs in the Fabaceae family contain pyrrolizidine alkaloids, which are associated with veno-occlusive liver disease [17]
Lamiaceae (5*)	*Ocimum gratissimum*	Mujaaja, Omujaaja (3)	African basil	Epigastric pain	Contains tannin phlobaphenes, flavones, flavonols, xanthones, chalcones, aurones, terpenes, flavononols, leucoanthocyanidins, catechins [39]	Herb from Ocimum genus (O. *lamiifolium*) was hepatotoxic in an *in-vitro* rat precision cut liver slice model [33], Ocimum *gratissimum* caused hepatoxicity *in-vivo* rabbit liver model [25]
	*Hoslundia opposita*	Kamunye (3)		To replace blood, postnatal health, vomiting during fever and jaundice	Contains sesquiterpenes and sesquiterpene alcohols [40]	

* one patient took both Ocimum gratissimum and Hoslundia oopposita. mg (milligrams), kg (kilograms).

**Table 4:** Characteristics of known herbs in remainder of plant families.

Family (n taking)	Scientific Name	Local Names (n taking)	English Name	Taken in Rakai for:	Known Pharmacology	Known Liver Toxicity and/or ART Interaction
Anacardiaceae (2)	*Rhus vulgaris or Rhus natalenis*	Olukansokanso, akakwansokwanso (1)		Antenatal health, aphrodisiac, gastrointestinal ulcers, back pain	No published information about contents	
	*Mangifera indica*	Mango Tree Bark (1)	Mango tree bark		Leaves contains flavonoids [41], Peel contains phenolic compounds and carotenoids [42]	
Primulaceae (2)	*Maesa lanceolata*	Oluwongwa, Oluwongo (2)		Neonatal jaundice	Contains Saponins [43], benzoquinone [44]	
Euphorbiaceae (2)	*Sapium ellipticum*	Musasa, Omusiisa, Musanvuma (2)	Jumping tree seed	Hypertension, antenatal health, sexually transmitted infections, epigastric pain	Contains phenols [45]	
Amaryllidaceae (1)	*Allium sativum*	Garlic (1)	Garlic	—	Contians diallyl disulfide [46]	Induces CYP3A4 and Pgp and should not be taken with the following antiretrovirals: APV, ATV, AZT, EFV, IDV, LPV, NFV, NVP, SQV [27]
Bignoniaceae (1)	*Spathodea campanulata*	Ekifabakazi (1)	African tulip tree	Dysmennorrhea, antenatal health	Contains 3β-acetoxyoleanolic acid, siaresinolic acid, oleanolic acid, others [47]	
Solanaceae (1)	*Solanum incanum*	Akatengotengo (1)	Sodom apple	Cough, chest pain	Contains alkaloids, saponins, solanine; High concentrations cause hemolysis of erythrocytes [48]	Herbs containing pyrrolizidine alkaloids are associated with veno-occlusive liver disease [17].
Vitaceae (1)	*Cyphostemma adenocaule*	Kamombo (1)		Peptic ulcers	Contain carotenoids (carotenes), xanthophylls, Vitamin C, Tocopherols, and Tocotrienols [49]	
Myrtaceae (1)	*Callistemon citrinus*	Bottlebrush (1)	Bottlebrush tree	Rhino-sinusitis	Contains 1,8-cineole, apha-pinene [50]	
		Kagulukandayi, Katangulucumu (1)		Epilepsy		
		Kakubamusolo (1)				

APV (amprenavir), ATV (atazanavir), AZT (zidovudine), EFV (efavirenz), IDV indinavir), LPV (lopinavir), NFV (nelfinavir), NVP (nevirapine), SQV (saquinavir).

### Herb Use and Liver Fibrosis

Among the 137/962 (14%) subjects with significant liver fibrosis, 12/137 (9%) reported herb use. Of the 825/962 (86%) subjects without significant liver fibrosis, 29/825 (4%) reported current herb use (p = 0.005). 56/494 (11%) of HIV-uninfected participants had significant fibrosis, compared to 81/468 (17%) of HIV-infected participants (p = 0.008).

In multivariable analysis that adjusted for age, fishing occupation, HIV infection, positive HBsAg, gender, and heavy liquor use, herb use was associated with two to five fold increases in significant liver fibrosis (see table 5). Among all participants, use of any herb (adjPRR = 2.2, 95% CI 1.3–3.5, p = 0.002), herbs from the Asteraceae family (adjPRR = 5.0, 95% CI 2.9–8.7, p<0.001), and herbs from the Lamiaceae family (adjPRR = 3.4, 95% CI 1.2–9.2, p = 0.017) were associated with increased significant fibrosis. Use of herbs from the Fabaceae family was not associated with significant liver fibrosis (adjPRR = 1.6, 95% CI 0.26–10.3, p = 0.60).

**Table 5:** Association of herbs with significant liver fibrosis in all participants.

	Univariate			Multivariate		
Herb (n taking)	PRR	95% CI	P value	adjPRR	95% CI	P value
Any current herb use (41)	2.2	1.3–3.6	0.003	2.2	1.3–3.5	0.002
Asteraceae (8)	5.5	3.6–8.4	<0.001	5.0	2.9–8.7	<0.001
Fabaceae (6)	1.2	0.19–7.1	0.86	1.6	0.26–10.3	0.60
Lamiaceae (5)	2.8	0.96.0–8.4	0.060	3.4	1.2–9.2	0.017
Unknown herb (21)	1.0	0.35–2.9	0.995	1.2	0.40–3.3	0.79

Multivariate model for all participants adjusts for: age, occupational fishing, HIV infection, positive Hepatitis B surface antigen, gender, heavy liquor use (≥1.25 L/week). Only participants with a valid TE scan (962/1000) were included in the model. CI (Confidence Interval).

Of 81 HIV-infected subjects with significant liver fibrosis, 4 (5%) reported herb use (see table 6). Among 387 HIV-infected subjects without significant liver fibrosis, 4 (1%) reported herb use (p = 0.014). In the multivariable analysis of HIV-infected participants adjusted for age, occupational fishing, positive HBsAg, gender, heavy liquor use, ART, and CD4 nadir, the associations between herb use and significant liver fibrosis were similar to findings among all participants. Among HIV-infected participants the use of any herb (adjPRR = 2.3, 95% CI 1.0–5.0, p = 0.044) and the use of herbs from the Asteraceae family (adjPRR = 5.0, 95% CI 1.7–14.7, p = 0.004) were associated with increased liver fibrosis.

**Table 6:** Association of herbs with significant liver fibrosis in HIV-infected participants.

	Univariate			Multivariate		
Herb (n taking)	PRR	95% CI	P value	adjPRR	95% CI	P value
Any current herb use (8)	3.0	1.4–6.2	0.003	2.3	1.0–5.0	0.044
Asteraceae (2)	6.0	4.9–7.3	<0.001	5.0	1.7–14.7	0.004
Unknown herb (5)	1.2	0.20–6.8	0.87	1.0	0.15–6.7	0.998

Multivariate model for HIV-infected participants adjusts for: age, occupational fishing, positive Hepatitis B surface antigen, gender, heavy liquor use (≥1.25 L/week), ART, and CD4 nadir. Only participants with a valid TE scan (468/500) were included in the model. CI (Confidence Interval).

Among all participants as well as HIV-infected participants, herb use was not associated with increased hepatotoxicity. 8/41 (20%) of participants reporting herb use had ACTG grade 1–4 ALT elevations, compared to 216/961 (23%) who did not report herb use (p = 0.56). Among HIV-infected participants reporting herb use, 6/33 (18%) had grade 1–4 ALT elevations, compared to 79/461 (17%) who did not report herb use (p = 0.88).

Table 7 shows the proportion of participants who took individual herbs in the Asteraceae, Lamiaceae, and Fabaceae families who had significant liver fibrosis. 6/8 participants taking herbs in the Asteraceae family had significant liver fibrosis. 4/6 subjects who used herbs in the *Vernonia* genus of the Asteraceae family had significant liver fibrosis.

**Table 7:** Use of specific herbs and significant liver fibrosis.

Herb used	Proportion of participants taking with significant liver fibrosis
**Asteraceae Family**	**6/8**
*Vernonia amygdalina*	2/4
*Vernonia*, species unknown	2/2
*Microglossa densiflora*	1/1
*Aspilia Africana*	1/1
**Fabaceae Family**	**2/6**
*Pseudarthria hookeri*	1/4
*Indigofera congesta*	1/2
**Lamaceae Family**	**2/5** *
*Ocimum gratissimum*	1/3
*Hoslundia opposita*	1/3

* One participant took both Ocmum gratissimum and Hoslundia opposite.

Only participants with a valid TE scans are shown.

## Discussion

This study indicates that traditional herbal medicine use may contribute to liver disease in Uganda. Use of traditional herbal medicines was independently associated with two to five fold increases in significant liver fibrosis. Herbs from the Asteraceae family were the most often used and showed the strongest association with significant liver fibrosis: a five-fold increase in all participants (p<0.001) and HIV-infected participants (p = 0.004).

Six of eight participants who took herbs in the Asteraceae family had significant liver fibrosis (see table 5). Many plants in the Asteraceae and Fabaceae families contain pyrrolizidine alkaloids, a known risk factor for veno-occlusive liver disease [7], [17]. Although none of the alkaloid-containing herbs used by participants in this study have been confirmed to contain pyrrolizidine alkaloids, ingestion of plants containing pyrrolizidine alkaloids caused outbreaks of veno-occlusive liver disease in Jamaica, India, Egypt, and South Africa [17], [18]. No outbreaks of veno-occlusive liver disease associated with pyrrolizidine alkaloids have been reported to our knowledge in East Africa. Pyrrolizidine alkaloids are inert until dehydrogenation by cytochrome P450 3A4 (CYP3A4) in the liver [19], where reactive toxic pyrrolic and N-oxide metabolites directly damage liver sinusoidal endothelial cells and hepatocytes (zone III of the liver acinus) [20]. Pyrroles cause chromosomal damage in a dose-dependent manner, resulting in an inflammatory response that culminates in fibrin deposition [17], [20], [21].

Although plants in both the Asteraceae and Fabaceae families ingested by study participants may contain pyrrolizidine alkaloids, our data shows a strong association between significant liver fibrosis and use of herbs in the Asteraceae family but not the Fabaceae family. The literature about African traditional herbal medicines is limited and does not explain why this difference might exist. Traditional herbal medicine remedies used in Rakai and throughout Uganda are often mixtures containing multiple herbs [8], [22]. It is possible that herbs in the Asteraceae family are taken at high doses, or potentiate the toxicity of other herbs or hepatotoxins.

Two participants with fibrosis reported use of *Vernonia amygdalina* in the Asteraceae family. This particular herb is commonly used in Africa is thought to have hepatoprotective properties [23]. However, animal studies show that at higher doses, this member of the Asteracaae family may be hepatotoxic. In an *in-vivo* rat CCl_4_ liver injury model, low doses (250–500 mg/kg) of *Vernonia amygdalina* were hepatoprotective, but a high dose (750 mg/kg) caused increased hepatoxicity [24].

Herbs from the Lamiaceae family were associated with a 3.4 fold increase in significant liver fibrosis among all participants in our study (p = 0.017). Herbs in the Lamiaceae family have been associated with hepatoxicity in an *in-vivo* rabbit model [25]. In addition, Aloe, taken by two participants in our study, has been linked in case reports to severe hepatitis [26]. However, data about the potential hepatotoxicity of many herbs used by participants in this study do not exist, or come from animal model studies only that should be interpreted cautiously.

The risk of significant fibrosis associated with herb use was similar in the overall and HIV- infected study populations. Data on herb use was limited in the HIV-infected population, and plant family specific analysis was only possible for the Asteraceae family. Only two HIV- infected participants reported using herbs in the Asteraceae family.

Despite the small number of HIV-infected participants in this study who reported herb use, it is important to note that ART may alter the toxicity profile of co-administered herbs. CYP3A4 is a major pathway for metabolism of a wide range of chemically distinct foreign compounds including phytochemicals and antiretroviral drugs [27]. Antiretroviral drugs of the non- nucleoside reverse transcriptase inhibitor (NNRTI) and protease inhibitor (PI) classes are also inducers or inhibitors of CYP3A4 activity [28], [29]. Therefore, these drugs have potential to influence phytochemical toxification or detoxification pathways in the liver. For example, commonly used NNRTI in initial ART regimens in Uganda (efavirenz and nevirapine) are inducers of CYP3A4 and therefore have potential to increase generation of toxic metabolites of pyrrolizidine alkaloids [27], [28]. Inhibitors of CYP3A4 may lead to accumulation of phytochemicals or their metabolites in the liver which may also result in toxicity.

Conversely, herbs may potentiate ART toxicities by influencing antiretroviral drug disposition in the liver, kidney, and gut. Herbs may affect NNRTI and PI metabolism by CPY3A4 and alter activity of cellular drug transporters and glucuronidation pathways [27]. Existing evidence from Africa about herb-ART interactions is limited to two herb families commonly used in South Africa: *Hypoxis* (African potato) and *Sutherlandia*, neither of which were taken by participants in this study. Hypoxis causes a dose-dependent inhibition of CYP3A4 up to 86% of the normal activity of CYP3A4 and 50% reduction of the expression of P-glycoprotein. Sutherlandia *frutescens* also causes a dose dependent inhibition of CYP3A4 up to 96% of CYP3A4 activity [30]. One participant in this study reported garlic use, which is known to significantly reduce concentrations of a PI (saquinavir), most likely by induction of CYP3A4 [31]. Since nevirapine and efavirenz are also eliminated by CYP3A4, garlic may reduce plasma levels of these drugs, but there are no clinical data on these interactions.

### Limitations

This study had limitations. The study was cross-sectional and only information about current herb use was available for analysis. Only 4% of participants in this study reported using herbs, compared to other studies in Uganda in which 60% of HIV-infected persons reported concurrent use of ART and herbs [2]. Some misclassification of herb exposure could have occurred due to a social desirability or reporting bias, especially among HIV-infected persons on ART who are counseled to avoid herbs in the communities around Rakai. Only 2% of HIV- infected participants reported herb use. While this lower number of HIV-infected participants reporting herb use could represent effective counseling, the difference in herb use among those on ART and those not on ART was not significant (1% vs. 2%, p = 0.42). The small number of participants reporting herb use limited many comparisons (e.g., herb-ART interactions) and suggests that our findings should be interpreted cautiously.

An important limitation of this study is the potential for reverse causality. Although the most frequently used families of herbs in this study contain known hepatotoxins (see table 3), it is possible that the association of fibrosis with herb use could represent reverse causality, or persons with symptomatic liver disease being more likely to use herbal medicines. According to consultations with local traditional practitioners, some of the herbs in Asteraceae and other families are sometimes prescribed for “fever with jaundice”. However, none of the study participants had been previously diagnosed with liver disease within the formal medical system or by traditional healers. Most herbs used in this study to treat fever are usually taken for general fever (“fever” or “malaria”), not fever with jaundice (“yellow fever”).

## Conclusions

More studies are needed to assess the impact of traditional herbal medicines in sub-Saharan Africa. Phytochemical, pharmacokinetic and prospective clinical studies are needed to investigate herb contents, benefits, side effects, direct toxicity, and herb-ART interactions. Plants in the Asteraceae family reported in this study should be prioritized for these investigations.

The risk of liver disease associated with herb use was similar in the overall study population and among HIV-infected participants. Given the potential of at least additive risk of hepatotoxicity with long term use of some antiretroviral drugs, as well as the potential for herbs to alter the pharmacology of antiretroviral drugs, it may be prudent to counsel HIV-infected persons against herb use in sub-Africa until there is data about the safety of specific herbs. Counseling about herb use should be part of routine health counseling and counseling of HIV- infected persons in sub-Africa.

## References

[1] Hamill FA, Apio S, Mubiru NK, Mosango M, Bukenya-Ziraba R, et al (2000) Traditional herbal drugs of southern Uganda, I. J Ethnopharmacol 70: 281–300. 10837990 10.1016/s0378-8741(00)00180-x

[2] Langlois-Klassen D, Kipp W, Rubaale T (2008) Who's talking? Communication between health providers and HIV-infected adults related to herbal medicine for AIDS treatment in western Uganda. Soc Sci Med 67: 165–176. 18406030 10.1016/j.socscimed.2008.02.027

[3] Tabuti J, Lye K, Dhillion S (2003) Traditional herbal drugs of Bulamogi, Uganda: plants, use, and administration. J Ethnopharmacol 88: 19–44. 12902048 10.1016/s0378-8741(03)00161-2

[4] Dara L, Hewett J, Lim JK (2008) Hydroxycut hepatotoxicity: a case series and review of liver toxicity from herbal weight loss supplements. World J Gastroenterol 14: 6999–7004. 19058338 10.3748/wjg.14.6999 PMC2773866

[5] Teschke R, Fuchs J, Bahre R, Genthner A, Wolff A (2010) Kava hepatotoxicity: comparative study of two structured quantitative methods for causality assessment. J Clin Pharm Ther 35: 545–563. 20831679 10.1111/j.1365-2710.2009.01131.x

[6] Ashafa AO, Sunmonu TO, Afolayan AJ (2010) Toxicological evaluation of aqueous leaf and berry extracts of Phytolacca dioica L. in male Wistar rats. Food Chem Toxicol 48: 1886–1889. 20450871 10.1016/j.fct.2010.04.029

[7] Asres K, Sporer F, Wink M (2007) Identification and quantification of hepatotoxic pyrrolizidine alkaloids in the Ethiopian medicinal plant Solanecio gigas (Asteraceae). Pharmazie 62: 709–713. 17944327

[8] Lamorde M, Tabuti JR, Obua C, Kukunda-Byobona C, Lanyero H, et al (2010) Medicinal plants used by traditional medicine practitioners for the treatment of HIV/AIDS and related conditions in Uganda. J Ethnopharmacol 130: 43–53. 20451595 10.1016/j.jep.2010.04.004

[9] Stabinski L, Reynolds S, Ocama P, Laeyendecker O, Ndyanabo A, et al (2011) High Prevalence of Liver Fibrosis Associated with HIV Infection: A Study in Rural Rakai, Uganda. Antiviral Therapy 16: 405–411. 21555823 10.3851/IMP1783 PMC3142695

[10] Wawer MJ, Gray RH, Sewankambo NK, Serwadda D, Paxton L, et al (1998) A randomized, community trial of intensive sexually transmitted disease control for AIDS prevention, Rakai, Uganda. AIDS 12: 1211–1225. 9677171 10.1097/00002030-199810000-00014

[11] AIDS Clinical Trials Group (1994) Table for grading the severity of adult and pediatric adverse advents. Rockville (Maryland): National Institutes of Health, Division of AIDS.

[12] Prati D, Taioli E, Zanella A, Della Torre E, Butelli S, et al (2002) Updated definitions of healthy ranges for serum alanine aminotransferase levels. Ann Intern Med 137: 1–10. 12093239 10.7326/0003-4819-137-1-200207020-00006

[13] Ruhl CE, Everhart JE (2012) Upper limits of normal for alanine aminotransferase activity in the United States population. Hepatology 55: 447–454. 21987480 10.1002/hep.24725 PMC3268908

[14] Andersen ES, Christensen PB, Weis N (2009) Transient elastography for liver fibrosis diagnosis. Eur J Intern Med 20: 339–342. 19524169 10.1016/j.ejim.2008.09.020

[15] Kirk GD, Astemborski J, Mehta SH, Spoler C, Fisher C, et al (2009) Assessment of liver fibrosis by transient elastography in persons with hepatitis C virus infection or HIV- hepatitis C virus coinfection. Clin Infect Dis 48: 963–972. 19236273 10.1086/597350 PMC2715996

[16] Barros AJ, Hirakata VN (2003) Alternatives for logistic regression in cross-sectional studies: an empirical comparison of models that directly estimate the prevalence ratio. BMC Med Res Methodol 3: 21. 14567763 10.1186/1471-2288-3-21 PMC521200

[17] Chen Z, Huo JR (2010) Hepatic veno-occlusive disease associated with toxicity of pyrrolizidine alkaloids in herbal preparations. Neth J Med 68: 252–260. 20558855

[18] Ridker PM, McDermott WV (1989) Comfrey herb tea and hepatic veno-occlusive disease. Lancet 1: 657–658. 2564469 10.1016/s0140-6736(89)92154-5

[19] Prakash AS, Pereira TN, Reilly PE, Seawright AA (1999) Pyrrolizidine alkaloids in human diet. Mutat Res 443: 53–67. 10415431 10.1016/s1383-5742(99)00010-1

[20] Chen T, Mei N, Fu PP (2010) Genotoxicity of pyrrolizidine alkaloids. J Appl Toxicol 30: 183–196. 20112250 10.1002/jat.1504 PMC6376482

[21] Yeong ML, Wakefield SJ, Ford HC (1993) Hepatocyte membrane injury and bleb formation following low dose comfrey toxicity in rats. Int J Exp Pathol 74: 211–217. 8499322 PMC2002119

[22] Tabuti J, Collins B, Kukunda C, Waako P (2010) Medicinal plants used by traditional medicine practitioners in the treatment of tuberculosis and related ailments in Uganda. J Ethnopharmacol 127: 130–136. 19799983 10.1016/j.jep.2009.09.035

[23] Iwalokun BA, Efedede BU, Alabi-Sofunde JA, Oduala T, Magbagbeola OA, et al (2006) Hepatoprotective and antioxidant activities of Vernonia amygdalina on acetaminophen- induced hepatic damage in mice. J Med Food 9: 524–530. 17201640 10.1089/jmf.2006.9.524

[24] Adesanoye O, Farombi E (2009) Hepatoprotective effects of Vernonia amygdalina (Asteraceae) in rats treated with carbon tertachloride. Exp Toxicol Pathol 62: 197–206. 19581077 10.1016/j.etp.2009.05.008

[25] Effraim K, Jacks T, Sodipo O (2002) Histopathological studies on the toxicity of Ocimum gratissimum leave extract on some organs of rabbit. African Journal of Biomedical Research 6: 21–25.

[26] Yang HN, Kim DJ, Kim YM, Kim BH, Sohn KM, et al (2010) Aloe-induced toxic hepatitis. J Korean Med Sci 25: 492–495. 20191055 10.3346/jkms.2010.25.3.492 PMC2826749

[27] van den Bout-van den Beukel CJ, Koopmans PP, van der Ven AJ, De Smet PA, Burger DM (2006) Possible drug-metabolism interactions of medicinal herbs with antiretroviral agents. Drug Metab Rev 38: 477–514. 16877262 10.1080/03602530600754065

[28] Barry M, Mulcahy F, Merry C, Gibbons S, Back D (1999) Pharmacokinetics and potential interactions amongst antiretroviral agents used to treat patients with HIV infection. Clin Pharmacokinet 36: 289–304. 10320951 10.2165/00003088-199936040-00004

[29] Dickinson L, Khoo S, Back D (2010) Pharmacokinetics and drug-drug interactions of antiretrovirals: an update. Antiviral Res 85: 176–189. 19665485 10.1016/j.antiviral.2009.07.017

[30] Mills E, Foster BC, van Heeswijk R, Phillips E, Wilson K, et al (2005) Impact of African herbal medicines on antiretroviral metabolism. AIDS 19: 95–97. 15627040 10.1097/00002030-200501030-00013

[31] Piscitelli SC, Burstein AH, Welden N, Gallicano KD, Falloon J (2002) The effect of garlic supplements on the pharmacokinetics of saquinavir. Clin Infect Dis 34: 234–238. 11740713 10.1086/324351

[32] Akah P, Okafor C (1992) Blood sugar lowering effect of Vernonia amygdalina (del) in an experimental rabbit model. Phytotherapy Research 6: 171–173.

[33] Mukazayire MJ, Allaeys V, Buc Calderon P, Stevigny C, Bigendako MJ, et al (2010) Evaluation of the hepatotoxic and hepatoprotective effect of Rwandese herbal drugs on in vivo (guinea pigs barbiturate-induced sleeping time) and in vitro (rat precision-cut liver slices, PCLS) models. Exp Toxicol Pathol 62: 289–299. 19493662 10.1016/j.etp.2009.04.005

[34] Tamokou JD, Kuiate JR, Tene M, Tane P (2009) Antimicrobial clerodane diterpenoids from Microglossa angolensis Oliv. et Hiern. Indian J Pharmacol 41: 60–63. 20336218 10.4103/0253-7613.51340 PMC2841233

[35] Kuiate J, Zollo PA, Lamaty G, Mentu C, Besseiere J (1999) Composition of essential oils from the leaves of two varieties of Aspilia africana (Pers) C.D Adams from Cameroon. Flavor and Fragrance 14: 167–169.

[36] Taziebou LC, Etoa FX, Nkegoum B, Pieme CA, Dzeufiet DP (2006) Acute and subacute toxicity of Aspilia africana leaves. Afr J Tradit Complement Altern Med 4: 127–134. 20162083 PMC2816443

[37] Njamen D, Magne Nde CB, Tanee Fomum Z, Vollmer G (2008) Effects of the extracts of some tropical medicinal plants on estrogen inducible yeast and Ishikawa screens, and on ovariectomized Wistar rats. Pharmazie 63: 164–168. 18380406

[38] Bakasso S, Lamien-Meda A, Lamien CE, Kiendrebeogo M, Millogo J, et al (2008) Polyphenol contents and antioxidant activities of five Indigofera species (Fabaceae) from Burkina Faso. Pak J Biol Sci 11: 1429–1435. 18817242 10.3923/pjbs.2008.1429.1435

[39] Matias EF, Santos KK, Almeida TS, Costa JG, Coutinho HD (2010) Phytochemical screening and modulation of antibiotic activity by Ocimum gratissimum L. Biomed Pharmacother 23: 23. 10.1016/j.biopha.2010.09.024 21115320

[40] Gundidza GM, Deans SG, Svoboda KP, Mavi S (1992) Antimicrobial activity of essential oil from Hoslundia opposita. Cent Afr J Med 38: 290–293. 1477878

[41] Kanwal Q, Hussain I, Latif Siddiqui H, Javaid A (2010) Antifungal activity of flavonoids 10.1080/14786419.2010.488628 21108117

[42] Ajila CM, Rao LJ, Rao UJ (2010) Characterization of bioactive compounds from raw and ripe Mangifera indica L. peel extracts. Food Chem Toxicol 48: 3406–3411. 20851730 10.1016/j.fct.2010.09.012

[43] Foubert K, Cuyckens F, Vleeschouwer K, Theunis M, Vlietinck A, et al (2010) Rapid quantification of 14 saponins of Maesa lanceolata by UPLC-MS/MS. Talanta 81: 1258–1263. 20441893 10.1016/j.talanta.2010.02.018

[44] Arot Manguro LO, Midiwo JO, Kraus W, Ugi I (2003) Benzoquinone derivatives of Myrsine africana and Maesa lanceolata. Phytochemistry 64: 855–862. 14559281 10.1016/s0031-9422(03)00428-x

[45] Adesegun SA, Elechi NA, Coker HA (2008) Antioxidant activities of methanolic extract of Sapium ellipticum. Pak J Biol Sci 11: 453–457. 18817172 10.3923/pjbs.2008.453.457

[46] Nahdi A, Hammami I, Brasse-Lagnel C, Pilard N, Hamdaoui MH, et al (2010) Influence of garlic or its main active component diallyl disulfide on iron bioavailability and toxicity. Nutr Res 30: 85–95. 20226993 10.1016/j.nutres.2010.01.004

[47] Ngouela S, Tsamo E, Sondengam BL (1988) Extractives from bignoniaceae: constituents of the stem bark of Spathodea campanulata. Planta Med 54: 476. 17265331 10.1055/s-2006-962516

[48] Beaman-Mbaya V, Muhammed SI (1976) Antibiotic action of Solanum incanum Linnaeus. 10.1128/aac.9.6.920 PMC429651 945715

[49] Al-Duais M, Hohbein J, Werner S, Bohm V, Jetschke G (2009) Contents of vitamin C, carotenoids, tocopherols, and tocotrienols in the subtropical plant species Cyphostemma digitatum as affected by processing. J Agric Food Chem 57: 5420–5427. 19480451 10.1021/jf9003626

[50] Oyedeji OO, Lawal OA, Shode FO, Oyedeji AO (2009) Chemical composition and antibacterial activity of the essential oils of Callistemon citrinus and Callistemon viminalis from South Africa. Molecules 14: 1990–1998. 19513000 10.3390/molecules14061990 PMC6254323

